# Caregiving and mental health among workers: Longitudinal evidence from a large cohort of adults in Thailand

**DOI:** 10.1016/j.ssmph.2016.01.004

**Published:** 2016-03-02

**Authors:** Vasoontara Yiengprugsawan, Liana Leach, Janneke Berecki-Gisolf, Hal Kendig, David Harley, Sam-ang Seubsman, Adrian C Sleigh

**Affiliations:** a National Centre for Epidemiology and Population Health and Department of Global Health, Research School of Population Health, The Australian National University, Canberra, Australia; b Centre for Research on Ageing, Health and Wellbeing, Research School of Population Health, The Australian National University, Canberra, Australia; c Monash Injury Research Institute, Monash University, Melbourne, Australia; d School of Human Ecology, Sukhothai Thammathirat Open University, Nonthaburi, Thailand

**Keywords:** Carers, Caregivers, Mental health, Work and health, Longitudinal data, Cohort study, Thailand

## Abstract

**Background:**

As people in middle and lower income countries live longer, more people become sick, disabled, and frail and the demand for family caregiving grows. Thailand faces such challenges. This study investigates the relationship between caregiving and mental health among workers drawn from a large longitudinal cohort of Thai adults.

**Methods:**

Participants were drawn from the Thai Health-Risk Transition Study, a cohort study since 2005 of distance-learning adult Open University students residing nationwide. Caregiving status and binary psychological distress outcome (score 19–30 on Kessler 6) were recorded in 2009 and 2013 among cohort members who were paid workers at both years (*n*=33,972). Multivariate logistic regression was used to estimate the relationship between four-year longitudinal caregiving status and psychological distress in 2013, adjusting for potential covariates.

**Results:**

Longitudinal analyses revealed the transitional nature of care with 25% exiting and 10% entering the caring role during the four-year follow-up. Based on multivariate logistic regression, 2009–2013 caregiving status was significantly associated with psychological distress. Cohort members transitioning into caregiving and those who were caregivers in both 2009 and 2013 had a higher risk for psychological distress than non-caregivers (Adjusted Odds Ratios 1.40 [1.02–1.96] and 1.64 [1.16–2.33], respectively).

**Conclusion:**

Our findings provide evidence on caregiving and associated risk for psychological distress among working Thais. This adds to the limited existing literature in middle-income countries and highlights the potential pressure among caregivers in balancing work and care while preserving their own mental health.

## Introduction

Family members frequently care for loved ones. Increasing life expectancy has made the need for such care greater. Most caregiving is informal and caregivers balance diverse responsibilities, including employment responsibilities, while attempting to maintain their own health and wellbeing. Our study objective is to investigate the relationship between caregiving and psychological distress among workers drawn from a large longitudinal cohort of Thai adults. Based on the 1990 Pearlins׳ conceptual model of caregivers׳ stress (Pearlin, Mullan, Semple, & Skaff, 1990), we hypothesized that with limited state-supported social welfare, this caregiving burden falls largely to family and that caregiving would be associated with psychological distress after taking into account covariates, including differences in workplace characteristics.

Previous research suggests that employment plays an important role in understanding the relationship between caregiving and mental health; given that most caregivers are employed and employment may either provide relief in terms of time away from the caregiving role or add to the overall burden of responsibilities. A search of the previous literature on the topic of ‘caregiving’, ‘work’, and ‘mental health’ in the past two decades found that empirical evidence on the intersection between work, care and mental health derives mainly from Western societies (Leach et al., 2010, Butterworth et al., 2010, Farfan-Portet et al., 2010, Berecki-Gisolf et al., 2008, Schneider et al., 2013, Lyonette and Yardley, 2006). Research conducted in Britain and Belgium demonstrates the importance of formal employment as a factor modifying the relationship between informal caregiving and adverse health outcomes (Farfan-Portet et al., 2010). An Australian study documents the adverse effects of transitioning into the provision of informal care on reduced labour force participation among middle-aged women (Berecki-Gisolf et al., 2008). A European-based economic study assessing the work–care relationship revealed gender differences in intentions to remain in formal employment. Among females and males, respectively, time demands associated with job change and physical care burden affected decisions to exit the labour market (Schneider et al., 2013). In the UK, work stress also predicted overall psychological distress among working caregivers after a one year follow-up (Lyonette & Yardley, 2006).

Further investigation shows some evidence of the interplay between care and mental health in Asia, but mainly in ageing populations. Among Hong Kong Chinese caregivers, family burden and caregiving have been shown to significantly impact on the mental health of caregivers (Wong, Tsui, Pearson, Chen, & Chiu, 2004). In Singapore, informal caregivers were also reported to have higher levels of depression and to have worse health outcomes than non-caregivers (Chan, Malhotra, Malhotra, Rush, & Ostbye, 2013). Less research has explored caregiving and mental health in the context of employment within Asia. However, one study from Japan showed that eldercare among employees was a significant risk for depression (Honda, Date, Abe, Aoyagi, & Honda, 2014), and another showed that the size of the social support network protected against physical and mental ill health among caregivers of disabled people (Arai, Nagatsuka, & Hirai, 2008). Many middle and lower income countries now need increased family caregiving due to rapid population ageing, but research examining the effects of caregiving on workers in middle-income Asian countries remains limited. Local data are needed to understand the effects of increased family caregiving within these specific contexts.

Our research focuses on a specific Asian country with rapid population ageing – Thailand. With projections that 23.1% and 37.1% of the population in 2025 and 2050, respectively, will be aged over 60 years (United Nations, Department of Economic and Social Affairs, Population Division (2015)), the need for informal caring will rise. Thailand has a strong Buddhist culture and a belief system supportive of care for family members. The current study aims to investigate the impact of caregiving on mental health among Thai workers, accounting for differences in relevant covariates such as job characteristics. This is one of the first population-based, longitudinal investigations of the interaction between caregiving and psychological distress among workers in Thailand. By identifying those at risk for adverse mental health consequences of caregiving, and the magnitude of the problem, this information can be used to devise and advocate for preventive measures such as respite care support.

## Methods

### Study population and data

Participants were drawn from a national cohort (Thai Cohort Study – TCS); cohort members are distance-learning students who resided nationwide and were enroled at the Sukhothai Thammathirat Open University when they responded to a 20-page baseline questionnaire in 2005 (*n*= 87,151) (Sleigh, Seubsman, & Bain, 2008). A four and eight year follow-up were conducted with a response rate of approximately 70% at each follow-up (*n*=60,569 in 2009 and *n*=42,785 in 2013). At 2005 baseline, median age was 29 years, roughly half the sample were females, and approximately half were urban residents (Sleigh et al., 2008).

### 2009–2013 caregiver status

At the 4-year follow up in 2009, participants were asked: “do you care for a sick or disabled family member?” – 27.5% were caregiving part-time and 6.6% full-time (Yiengprugsawan, Harley, Seubsman, & Sleigh, 2012). ท่านต้องดูแลสมาชิกในครอบครัว หรือคนที่รู้จักที่ป่วย/ ทุพพลภาพ หรือไม่

At the 8-year follow-up in 2013, participants were asked: “do you care for a chronically ill, disabled, or frail family members?” “How many hours per week do you provide care?” “How many years have you cared for that person?” What types of care do you provide?” Responses include help with: mobility (moving the person); cognitive care; bath and/or get dressed; prepare or eat food; attend religious activities; emotional support; shopping; or financial support. ท่านต้องดูแลสมาชิกในครอบครัว หรือคนที่รู้จักที่ป่วยเรื้อรัง/ ทุพพลภาพ/ชราภาพ หรือไม่

Based on the longitudinal caregiving status provided in 2009 and 2013, the cohort was categorized into four groups: non-caregivers at both time points; caregivers in 2009 (but not in 2013); caregivers in 2013 (but not in 2009); and caregivers at both time points.

### Outcome and potential covariates

We used the Kessler 6 psychological distress scale, measured in both 2009 and 2013, as the primary outcome of the study: “in the past 4 weeks, how much of the time did you feel… (1) so sad nothing can cheer you up; (2) nervous; (3) restless or fidgety; (4) hopeless; (5) everything was an effort; (6) worthless. Five-point scale responses ranged from ‘all the time’ to ‘none of the time’”. Participants who scored ≥19 out of 30 were classified as having ‘high psychological distress’ (Kessler et al., 2002). The Kessler 6 has previously been applied in Asia (Oshio, 2014) and validated to have high reliability and validity in the Thai context (Suraaroonsamrit and Arunpongpaisal, 2014, Yiengprugsawan et al., 2015).

Other covariates in 2013 included:

– Demography: age, sex, marital status, household size, personal monthly income, urban–rural residence.
– Work: occupation groups, weekly paid work hours, job security (‘not at all’, ‘moderate’, ‘very secure’)
– Social support: “how much support do you feel you get from … family, neighbours, colleagues or supervisors?” (‘a little’, ‘somewhat’, ‘a lot’, ‘not applicable’)
– Satisfaction with spare time: “how satisfied are you with…. amount of spare time?” 0 (completely dissatisfied) to 10 (completely satisfied). Those scored 0–4 were classified as ‘not satisfied’, 5–7 as ‘somewhat satisfied’, and 8–10 as ‘very satisfied’.

### Statistical analyses

Descriptive analyses include the distribution of cohort characteristics and their caregiving status. Individuals with missing data for given analyses were excluded (<5% of all samples and not statistically different by age and sex groups). We restricted the analyses to paid workers in both 2009 and 2013, as a result, 33,972 cohort members were included in the analyses.

First, we describe 2013 caregiving activities by key demographic attributes. As the Kessler 6 psychological distress measure is commonly used as a binary outcome, multivariate logistic regression was used to estimate the relationship between four-year longitudinal caregiving status and psychological distress in 2013, adjusting for potential covariates. The results are presented in two models: the first model adjusts for 2009 baseline psychological distress scores and covariates; the second model is a restricted version of the first model and includes only cohort members who did not have ‘high’ psychological distress cases at 2009 baseline. Adjusted odds ratios and 95% confidence intervals are reported.

## Results

Among Thai cohort members at the 2009 and 2013 follow-up, 56.1% reported they were not caregivers in either year, 24.5% reported being caregivers in 2009 only, 8.6% in 2013 only, and 10.6% reported being caregivers at both time points (Table 1). Caregivers tended to be older and to reside in rural areas. The least frequent caregiving activities were ‘bathing and dressing’ (3.5%) and ‘help with mobility’ (3.7%) and the most common were ‘help shopping’ (14.1%) and ‘financial support’ (15.2%). Females, older cohort members, and rural residents reported higher caregiving activities.

**Table 1:** Caregiving status and related activities, Thai Cohort Study 2009 and 2013.

**Caregiving status and psychological distress prevalence**	**%(*****N*****=33,972)**^a^	**Cohort attributes (Column%)**						**Prevalence**^b^**of high psychological distress (Kessler 6)**	
		**Sex**		**Age groups**		**Residence**			
		Male	Female	<40 yrs	≥40 yrs	Rural	Urban		
		(15,220)	(18,752)	(17,561)	(16,411)	(14,905)	(18,830)	**2009**	**2013**
**2009–2013 caregiving status**									
No–No (non-caregivers both periods)	56.1	55.4	56.7	61.7	50.2	52.1	59.2	7.2	9.4
Yes–No (caregivers in 2009 only)	24.5	27.2	22.4	22.4	26.9	26.1	23.3	8.9	10.6
No–Yes (caregivers in 2013 only)	8.6	7.6	9.4	7.9	9.5	9.2	8.1	8.5	12.5
Yes–Yes (caregivers in both periods)	10.6	9.7	11.4	8.0	13.5	12.4	9.3	11.1	13.8
**Caregiving activities, 2013**									
Hours of care per week									
<10	4.7	4.6	4.8	3.7	5.8	5.3	4.3		
10–19	3.9	3.6	4.1	3.4	4.5	4.8	3.2		
20–34	3.5	3.0	3.9	2.9	4.2	4.1	3.1		
35+	4.8	3.6	5.8	3.9	5.8	5.6	4.2		
Years spent caring									
0–4 years	7.1	6.1	7.8	6.6	7.7	7.8	6.5		
5–10 years	7.8	7.2	8.4	6.7	9.1	9.1	6.9		
10+ years	3.9	3.3	4.4	2.2	5.8	4.4	3.6		
Caregiving activities^c^									
Help with mobility	3.7	3.3	4.1	3.1	4.5	4.1	3.5		
Help with cognitive care	4.8	3.8	5.7	3.4	6.5	5.5	4.4		
Help bathing and dressing	3.5	2.1	4.7	2.7	4.4	3.8	3.4		
Help preparing or eating food	6.1	4.2	7.8	4.8	7.7	7.1	5.5		
Help with religious activities	6.9	6.1	7.5	5.2	8.7	8.2	5.9		
Emotional support	8.5	6.9	9.8	6.9	10.2	9.9	7.4		
Help shopping	14.1	11.7	16.1	11.7	16.7	16.4	12.4		
Financial support	15.2	13.2	16.9	12.3	18.4	17.3	13.7		

a Percent indicates proportion of 2013 longitudinal cohort members who were in paid work in both 2009 and 2013 (*N*=33,972).

b Row percent.

c Multiple answers allowed.

Prevalence of high psychological distress is reported in Table 1 for both 2009 and 2013 by caregiving status. Non-caregivers in both periods had the lowest percentage of high psychological distress (7.2% in 2009 and 9.4% in 2013) compared to cohort members who had taken up caregiving by 2013 (8.5% and 12.5%) and those who were providing caregiving at both time points (11.1% and 13.8%).

We report adjusted odds ratios and 95% Confidence Intervals for high psychological distress in 2013, based on multivariate logistic regression (Table 2). After adjusting for potential covariates, 2009–2013 caregiving status was significantly associated with high psychological distress. Model 1 shows Adjusted Odds Ratios (AOR) of 1.26 for ‘new’ caregivers in 2013 compared to those who were never caregivers (but not statistically significant). Model 1 also shows AOR of 1.38 higher risk among those who were caregivers in both 2009 and 2013, compared to those who were never caregivers. After restricting the analyses to those without psychological distress in 2009 (Model 2), the adjusted caregiver effects were 1.40 and 1.64, respectively. Cohort members who no longer reported providing care (only caregiver in 2009) had slightly higher odds of high psychological distress compared to the non-caregivers (statistically significant in Model 2). Being older, unpartnered, and having lower income were associated with high reported psychological distress in 2013. Low job security, low social support (notably from colleagues or supervisors), and dissatisfaction with leisure time were strongly associated with high psychological distress.

**Table 2:** Longitudinal caregiving and psychological distress among workers, Thai Cohort Study 2009–2013.

**Cohort characteristics (column %,*****n*****=**33,972**)**	**Multivariate logistic adjusted Odds Ratios [95% confidence intervals]**	
	**Model 1** (adjusted for covariates and 2009 baseline psychological distress scores) *N*=31,243	**Model 2** (Model 1, restricted to 2009 baseline non-psychological distress cases) *N*=28,904
**Caregiver status 2009–2013**		
No–No (non-caregivers in both periods, 56.1%)	1.00	1.00
Yes–No (caregivers in 2009 only, 24.5%)	1.06 [0.96–1.17]	1.12 [0.99–1.26]
No–Yes (caregivers in 2013 only, 8.6%)	1.26 [0.94–1.69]	**1.40** [1.02–1.96]
Yes–Yes (caregivers in both periods, 10.6%)	**1.38** [1.01–1.88]	**1.64** [1.16–2.33]
**Caregiving intensity in 2013**		
*Hours of caregiving*		
Non-caregivers (83.1%)	1.00	1.00
<10 hours (4.7%)	1.19 [0.89–1.77]	1.19 [0.82–1.74]
10–19 hours (3.9%)	1.14 [0.94–1.88]	1.07 [0.72–1.58]
20–34 hours (3.5%)	1.02 [0.85–1.80]	1.00 [0.67–1.49]
35+ hours (4.8%)	1.08 [0.77–1.74]	0.98 [0.66–1.44]
*Years of caregiving*		
Non-caregivers (81.0%)	1.00	
0–4 years (7.1%)	0.87 [0.65–1.17]	0.83 [0.59–1.17]
5–10 years (7.9%)	0.88 [0.65–1.19]	0.79 [0.55–1.12]
10+ years (3.9%)	0.87 [0.62–1.21]	0.78 [0.53–1.14]
**Demographic attributes in 2013**		
Female (55.2%) – male as reference	1.06 [0.97–1.16]	1.03 [0.94–1.14]
*Age groups:*		
<40 years (51.6%)	1.00	1.00
40–49 years (34.3%)	**1.29 [1.11–1.53]**	**1.24** [1.03–1.48]
50+ years (13.9%)	**1.36 [1.01–1.82]**	1.23 [0.94–1.75]
*Marital status:*		
Married (64.0%)	1.00	1.00
Divorced, widowed, separated (8.2%)	**1.56 [1.36–1.79]**	**1.51 [1.29–1.77]**
Never married (27.7%)	**1.20 [1.09–1.33]**	**1.15 [1.03–1.28]**
*Household size:*		
1–2 (21.1%)	1.00	
3–4 (47.4%)	0.99 [0.89–1.12]	1.07 [0.95–1.21]
5+ (31.4%)	0.96 [1.09–1.33]	0.96 [0.87–1.07]
*Residence:*		
Rural (44.2%) – urban as reference	1.03 [0.94–1.12]	1.07 [0.97–1.18]
**Socioeconomic attributes in 2013**		
*Personal monthly income*		
<10,000 Baht (15.4%)	**1.39 [1.18–1.65]**	**1.43 [1.19–1.73]**
10,001–30,000 Baht (40.2%)	**1.33 [1.15–1.52]**	**1.33 [1.13–1.55]**
20,001–30,000 Baht (23.9%)	**1.28 [1.11–1.48]**	**1.28 [1.09–1.50]**
>30,000 Baht (20.2%)	1.00	1.00
*Occupation*		
Professionals/managers (44.1%)	1.00	1.00
Office assistants (34.8%)	**1.19** [1.08–1.31]	**1.20** [1.08–1.34]
Skilled/manual workers (17.1%)	1.10 [0.97–1.25]	1.11 [0.97–1.28]
Others (3.8%)	1.22 [0.77–1.93]	1.15 [0.67–1.98]
**Subjective social attributes in 2013**		
*Job security*		
Not at all (6.5%)	**2.19** [1.83–2.63]	**2.37** [1.94–2.91]
Moderately secure (66.1%)	**1.32** [1.17–1.49]	**1.36** [1.19–1.55]
Very secure (27.3%)	1.00	1.00
*Reported low social support from:*		
Family (low 12.1%)	**1.64** [1.47–1.83]	**1.74** [1.56–1.94]
Neighbours (low 14.2%)	**1.21** [1.06–1.38]	**1.18** [1.01–1.37]
Colleagues and supervisors (low 27.3%)	**1.65** [1.49–1.82]	**1.73** [1.54–1.97]
*Satisfaction with spare time*		
Not very satisfied (12.2%)	**3.97** [3.54–4.46]	**4.26** [3.74–4.86]
Somewhat satisfied (37.8%)	**2.16** [1.96–2.38]	**2.31** [2.07–2.58]
Very satisfied (49.9%)	1.00	1.00

## Discussion

This study investigated the association between caregiving and mental health among a large nationwide cohort of working Thai adults. All cohort members were in paid work and close to one fifth were caregivers with half providing 20 h or more care per week. Longitudinal analyses showed a relationship between changes in caregiving status and high psychological distress and revealed the transitional nature of caregiving (during 4-year follow-up a quarter of the cohort stopped caregiving and 10% started). Associations between caregiving and poor mental health remained after adjusting for job characteristics and other relevant covariates.

Reported caregiving fell between 2009 and 2013 (approx. 35–20%). This could mean: (1) care recipients recovered or died; (2) cohort members finished their Open University degree and were no longer available for caregiving; (3) the 2009 question included part-time care as a specific category and 2013 did not – as a result some 2013 part-time caregivers may have answered ‘no’. Most of our caregivers were part-time or transient (i.e. different results in 2009 and 2013) but our study still captured a substantial number of long-term caregivers (i.e. 5% reported providing care for 5–10 years and 2.3% for more than 10 years).

Our findings support previous studies revealing the adverse impact of caregiving on mental health among workers (Pearlin et al., 1990, Farfan-Portet et al., 2010, Berecki-Gisolf et al., 2008, Lyonette and Yardley, 2006, Wong et al., 2004, Oshio, 2014). In addition, the analyses showed that several other important covariates were also associated with psychological distress. After mutually adjusting for demographic and work characteristics, job security was also strongly associated with psychological distress among the Thai cohort members. This supports our previous findings on the importance of job characteristics, especially perceived low job security, on mental health. Another important factor could be limited social welfare in middle-income settings such as Thailand (Yiengprugsawan et al., 2015). Cohort members with lower income were more likely to report psychological distress potentially due to limited financial resources. Lack of social support, particularly in the workplace, was also found to be a risk factor for high psychological distress. Similar findings have been reported in other studies examining the importance of support from colleagues and supervisors (Wong et al., 2004, Yiengprugsawan et al., 2015). Workplace flexibility is known to alleviate work-family conflict (Li, Shaffer, & Bagger, 2015). This study found that satisfaction with spare time was associated with reduced psychological distress; time pressure has previously been shown to affect the mental health of caregivers (Brown and Pitt-Catsouphes, 2013).

The main strength of this study lies in the large nationwide longitudinal cohort of Thais who provided information on caregiving status, mental health, as well as detailed work and social characteristics, enabling investigation of the links between caregiving and mental health. Although cohort members were similar to the Thai population in terms of their median income and geographical residence, they were enroled as distance-learners in open-university education and hence were more likely to have completed high-school than other Thais. While the sample is not necessarily representative of all caregivers, it does provide insight into the upwardly mobile, educated group of modest means who arguably are at the forefront of social change in Thailand.

We also note some limitations related to our data. First, the caregiving question in 2009 captured ‘sick’ and ‘disabled’ family members, however in 2013 this question was rephrased and captured ‘chronically ill’, ‘disabled’, but also ‘frail’ family members. However, it should be noted that the English translation for the caregiving questions between 2009 and 2013 suggests greater disparity than the Thai translation, in which the word for ‘frail’ (ชราภาพ) is closely tied in meaning to sickness or disability (ป่วย/ ทุพพลภาพ). Therefore in Thai, the disparity in meaning between the two surveys is less than in English. Comparisons in caregiver status and ‘years caregiving’ between the 2009 and 2013 surveys suggest the caregiving role was interpreted similarly despite differences in terminology. Second, we did not gather information on the relationship between caregiver and recipient in 2013. Although, we did record caregiving activities, length of care, and time spent caring, and so were able to gain an understanding of care intensity. In 2012, phone interviews were conducted with a subsample of caregivers (*n*=115) and results showed that about 60% (*n*=68) reported caring for parents and 15% (*n*=17) cared for grandparents. Third, we noted approximately 30% attrition between 2009 and 2013. However, when comparing those who dropped-out (*n*=17,453) to those who did not (*n*=43,116) the proportion of caregivers in 2009 were similar (26.2% full-time and 5.7% part-time caregivers vs 28.1% vs 6.9%). Further qualitative investigation could provide insight into the needs of caregivers and may help design interventions to improve their mental health. Detailed information on the nature of work, social support and leisure time is not available longitudinally in this study. Future follow-up could repeat the key caregiving status and mental health measures to monitor medium and long-term relationships between caregiving and mental health among Thai workers.

Our findings highlight the frequently transitional nature of caregiving amongst Thai workers, and the substantial adverse impacts on mental health. Balancing work and care is likely to be vital for many to remain in the workforce, which is important for financial and social reasons. Protecting caregivers׳ mental health could potentially benefit the quality of care, and avoid future adverse health outcomes such as caregivers themselves becoming ill, and/or reductions in workforce participation. The significance of perceived job security, support at work, and leisure time warrants policy responses to facilitate workplace flexibility and options such as respite care and carer׳s leave. This may assist caregivers in combining their work with caring responsibilities, without compromising their mental health.

## Funding

This study was supported by the International Collaborative Research Grants Scheme with joint Grants from the Wellcome Trust UK (GR071587MA) and the Australian National Health and Medical Research Council (268055), and as a global health Grant from the NHMRC (585426).
